# Socioeconomic inequality in hospital case fatality rate and care among children and adolescents hospitalized for COVID-19 in Brazil

**DOI:** 10.1590/1980-549720230015

**Published:** 2023-02-20

**Authors:** Caroline Fabrin, Alexandra Crispim Boing, Leandro Pereira Garcia, Antonio Fernando Boing

**Affiliations:** I Universidade Federal de Santa Catarina, Graduate Program in Collective Health – Florianópolis (SC), Brazil.; II Florianópolis Municipal Health Department – Florianópolis (SC), Brazil.

**Keywords:** COVID-19, Child, Mortality, Hospitalization, Health status disparities, COVID-19, Criança, Letalidade, Hospitalização, Desigualdade em saúde

## Abstract

**Objective:**

To analyze the association of hospital case fatality rate and care received by children and adolescents hospitalized for COVID-19 with the gross domestic product (GDP) *per capita* of Brazilian municipalities and regions of residence.

**Methods:**

Data were collected from the Influenza Epidemiological Surveillance Information System and the Brazilian Institute of Geography and Statistics. The dichotomous outcomes analyzed were hospital case fatality rate of COVID-19, biological samples collected for COVID-19 diagnosis, X-rays, computed tomography (CT) scans, use of ventilatory support, and intensive care unit hospitalization. The covariates were municipal GDP *per capita* and the Brazilian region of residence. Poisson regression was used for the outcomes recorded in 2020 and 2021 in Brazil, covering the two COVID-19 waves in the country, adjusted for age and gender.

**Results:**

The hospital case fatality rate was 7.6%. In municipalities with lower GDP *per capita* deciles, the case fatality rate was almost four times higher among children and twice as high in adolescents compared to cities with higher deciles. Additionally, residents of municipalities with lower GDP *per capita* had fewer biological samples collected for diagnosis, X-ray examinations, and CT scans. We found regional disparities associated with case fatality rate, with worse indicators in the North and Northeast regions. The findings remained consistent over the two COVID-19 waves.

**Conclusion:**

Municipalities with lower GDP *per capita*, as well as the North and Northeast regions, had worse indicators of hospital case fatality rate and care.

## RESUMO

**Objetivo:**

Analisar a associação entre a letalidade e o cuidado hospitalar recebido por crianças e adolescentes internados por COVID-19 e o produto interno bruto (PIB) *per capita* dos municípios brasileiros e a região de residência.

**Métodos:**

Os dados foram extraídos do Sistema de Informação de Vigilância Epidemiológica da Gripe e do Instituto Brasileiro de Geografia e Estatística. Analisaram-se como desfechos dicotômicos a letalidade hospitalar por COVID-19, a coleta de amostra biológica para diagnóstico de COVID-19, a realização de exames raio X e tomografia, o uso de suporte ventilatório e a internação em unidade de terapia intensiva. As covariáveis foram o PIB municipal *per capita* e a região brasileira de residência. Foi realizada regressão de Poisson para os desfechos registrados em 2020 e 2021 no Brasil e segundo o período compreendido em duas ondas de COVID-19 no país, ajustando-a por idade e sexo.

**Resultados:**

A letalidade hospitalar foi de 7,6%. Nos municípios dos menores decis de PIB *per capita* a letalidade foi quase quatro vezes maior entre crianças e duas vezes mais elevada entre adolescentes quando comparada àquela dos maiores decis. Adicionalmente, os residentes de municípios com menor PIB *per capita* realizaram menos coleta de amostra biológica para diagnóstico, exames de raio X e tomografias. Foram encontradas disparidades regionais associadas à letalidade, com piores indicadores nas regiões Norte e Nordeste. Os achados mantiveram-se consistentes durante as duas ondas de COVID-19.

**Conclusão:**

Em municípios com menor PIB *per capita* e das regiões Norte e Nordeste houve piores indicadores de letalidade e cuidado hospitalar.

## INTRODUCTION

The COVID-19 pandemic has caused major disruptions in the lives of billions of people and health services worldwide. Studies have shown that these impacts had different intensities and durations according to the socioeconomic and environmental conditions of locations and individuals^1–4^, with worse outcomes for more disadvantaged groups^5^. COVID-19 is also the result of the interaction of social and economic inequalities with the distribution of pre-existing disease burden, described as a syndemic with the potential to further exacerbate these differences between groups^5^.

COVID-19 can cause severe conditions regardless of the infected person's age. Compared to older adults and adults, children and adolescents usually have a milder disease course and lower case fatality rate^6–11^; however, severe and potentially fatal acute and chronic manifestations, such as multisystem inflammatory syndrome, have been identified in this group^12,13^. In addition, the country experienced periods of high hospitalization rates, particularly in times of greater spread of the Delta and Omicron variants^14–16^. Also, a systematic review suggests that the COVID-19 impact on this younger age group is unevenly distributed among countries, both in admissions to intensive care units (ICU) and deaths^10^. Results of the study by Kitano et al.^10^ showed that more than 90% of deaths reported worldwide occurred in middle- and low-income countries, while ICU admissions corresponded to less than 30% in these countries.

COVID-19 also had a heavy impact on Brazilian children. It represented the second cause of death in the age group from 5 to 11 years in 2020^17^, and by December of the same year, the country had the highest mortality rate in the population up to 19 years, with 23.6 deaths per one million children^10^. In the same period, the United States presented a mortality rate 12 times lower^10^. In the United Kingdom, during the first year of the pandemic, the mortality rate was 1.4 deaths per one million children^10^, while in Colombia and Mexico, the rates were 7.8 and 5.4, respectively^10^.

The distribution of COVID-19 deaths among children and adolescents according to personal socioeconomic or residence conditions is still scarcely described^4,10,18,19^. Studies have shown that the COVID-19 case fatality rate in pediatric patients was associated with ethnicity and populations living in middle- and low-income countries. In sub-national analyses, deaths have been more frequent in the North and Northeast regions of Brazil^4,20–22^, but the investigations focused on the first year of the pandemic and did not analyze all COVID-19 cases recorded in the country in later periods. Also, we found no pediatric studies exploring inequalities in hospital care and case fatality rate in Brazilian municipalities in 2020 and 2021 and according to COVID-19 waves.

The severity of the pandemic originates from and also exacerbates social inequalities^5^. Brazil has considerable socioeconomic and regional disparities and presented an inadequate response to the pandemic^23,24^. In this context, knowing the outcomes of severe COVID-19 cases and the care provided to a vulnerable population is essential to help assess the actions taken and to serve as a basis for ongoing actions to combat COVID-19 and potential future epidemics.

This study aimed to analyze hospital case fatality rate, the proportion of patients tested for the disease, the use of imaging tests and ventilatory support, and the proportion of ICU hospitalizations for COVID-19 among children and adolescents according to the gross domestic product (GDP) *per capita* of their municipalities of residence and Brazilian macro-regions.

## METHODS

We analyzed data from the Influenza Epidemiological Surveillance Information System (*Sistema de Informação da Vigilância Epidemiológica da Gripe* — SIVEP-Gripe) and the Brazilian Institute of Geography and Statistics (*Instituto Brasileiro de Geografia e Estatística* — IBGE). SIVEP-Gripe is the official Brazilian system for the record of severe acute respiratory syndrome (SARS) cases and deaths. Reporting the cases is compulsory, even those caused by SARS-CoV-2.

The study population comprised children (0 to 11 years) and adolescents (12 to 18 years) hospitalized for SARS with final COVID-19 classification and whose symptoms started between epidemiological week 10 of 2020 and week 52 of 2021, according to the database updated on January 18, 2022. We excluded from the study 2,486 individuals whose report forms did not show the case progress (cure/death) and another 1,519 whose variable was filled as ignored or death from other causes. Another 5,025 individuals were also excluded because their report forms had ignored entries for study variables. Thus, we analyzed data from 22,610 COVID-19 hospitalizations.

The study outcomes were:

- Hospital case fatality rate of COVID-19;
- Biological samples collected to diagnose the etiologic agent;
- X-ray examinations;
- Computed tomography (CT) scans;
- Ventilatory support (invasive or non-invasive);
- ICU hospitalization.

All cases were classified dichotomously and analyzed according to the municipality of residence. The independent study variables were the municipal GDP *per capita* and the geopolitical macro-region of residence. The Brazilian municipalities were grouped into deciles based on their respective GDP *per capita* values. These values correspond to 2018; were calculated by IBGE for each municipality in partnership with state statistical agencies, state government departments, and the Superintendence of the Manaus Free Trade Zone; and are expressed in reais (Supplementary Table 3). The country's division into macro-regions was elaborated in 1970, resulting in the following denominations: North Region, Northeast Region, Southeast Region, South Region, and Midwest Region.

Relative risks (RR) were calculated for each outcome during the study period and for the two COVID-19 waves. The first wave comprised the period between epidemiological weeks 10 and 43 of 2020 and was marked by an expressive number of simultaneous cases in different regions of the country. The second wave occurred over the epidemiological weeks 44 of 2020 and 21 of 2021 and was associated with the spread of new variants, putting more pressure on an already overloaded health system, as described by Bastos et al.^25^. We used Poisson regression to estimate the RR and their respective 95% confidence intervals (95%CI), adjusting the values for gender and age. Outcome values were also expressed as percentages according to independent variables. Analyses were carried out in the R statistical software, version 4.0.2. The data are public and anonymized; thus, the study did not require approval by the Research Ethics Committee.

## RESULTS

Children or adolescents were hospitalized for COVID-19 in 3,138 of the 5,570 Brazilian municipalities between 2020 and 2021. The study included 22,610 hospitalizations for SARS with final COVID-19 classification. Most patients were in the age group of 0–11 years (72%). The hospital case fatality rate was 7.6%, 29.9% of the individuals were admitted to ICU beds, and just over half (52.8%) used ventilatory support. Biological samples were collected for diagnosis in 96.3% of cases. X-rays and CT scans were performed in 35.7 and 17% of cases, respectively (Table 1 and Supplementary Table 1).

**Table 1:** Sample description, proportion of biological samples collected, X-rays, computed tomography scans, ventilatory support, intensive care unit hospitalization, and case fatality rate in children hospitalized for COVID-19, according to gross domestic product *per capita* deciles of municipalities and macro-regions. Brazil, March 2020 to December 2021.

		Sample (n)	Biological samples collected (%)	X-ray examinations (%)	Computed tomography scans (%)	Ventilatory support (%)	ICU hospitalization (%)	Case fatality rate (%)
Income decile								
	1 (poorest)	367	92.6	38.1	5.7	51.5	28.6	15.5
	2	522	88.9	39.7	10.9	54	26.6	12.1
	3	476	93.7	43.3	6.3	54.8	30.9	10.9
	4	708	90.1	38	8.6	48.4	27.1	9.3
	5	1,138	96.4	39.3	7.6	47.5	26.7	7.6
	6	1,042	96.7	38.6	10.5	51	31.7	8.5
	7	2,027	96.4	34.1	9.6	45.6	30.7	6.7
	8	1,461	97.1	38.6	16.5	53.3	29.6	8.1
	9	2,743	96	38	11.3	49.9	26.1	4.1
	10 (richest)	5,799	98.4	44.1	11.8	54.7	32.1	4.1
Region								
	Northeast	3,492	94.7	33.6	7.7	51.5	34.7	11.1
	North	2,331	91.5	38.6	6.7	39.9	15.8	6.7
	Midwest	1,360	98.0	39.2	16.0	49.6	32.8	5.2
	South	2,039	98.9	44.5	12.7	53	24.2	4.5
	Southeast	7,061	97.9	42.7	12.6	55.3	33	4.4
	Total sample	16,283	96.5	40.1	11	51.5	29.8	6.3

ICU: intensive care unit.

The hospital case fatality rate of COVID-19 varied based on municipal GDP *per capita* deciles, reaching 15.5% among children living in poorer municipalities and 4.1% among inhabitants of richer ones. For adolescents, this variation was 16.2 and 8.8%. When analyzing the remaining outcomes, we noted that the proportions of patients with a biological sample collected for diagnosis, X-ray examinations, CT scans, ventilatory support, and ICU hospitalization were lower in municipalities with lower GDP *per capita* in both age groups. The Northeast Region presented the highest case fatality rate, and proportionally, the North Region had fewer biological samples collected for diagnosis, CT scans, use of ventilatory support, and ICU hospitalization (Table 1 and Supplementary Table 1).

Residents of poorer municipalities showed hospital case fatality rates almost four times higher among children and almost twice as high in adolescents compared to those living in richer cities (Table 2 and Supplementary Table 2). In addition, richer municipalities presented a higher proportion of patients with biological samples collected to diagnose the etiologic agent in both children and adolescents and 16% more (RR=1.16; 95%CI 1.01–1.32) X-ray examinations in children. The same phenomenon was identified for CT scans, which were twice as frequent among children and adolescents living in richer municipalities. We found no differences in the remaining outcomes (Table 2 and Supplementary Table 2).

**Table 2:** Relative risk of biological samples collected, X-rays, computed tomography scans, ventilatory support, intensive care unit hospitalization, and case fatality rate in children hospitalized for COVID-19, according to gross domestic product *per capita* deciles of municipalities and macro-region deciles. Brazil, March 2020 to December 2021.

		Biological samples collected (95%CI)	X-ray examinations (95%CI)	Computed tomography scans (95%CI)	Ventilatory support (95%CI)	ICU hospitalization (95%CI)	Case fatality rate (95%CI)
Income decile*							
	1 (poorest)	1.00	1.00	1.00	1.00	1.00	1.00
	2	0.96 (0.92–1.00)	1.04 (0.88–1.23)	1.85 (1.14–2.98)	1.05 (0.92–1.19)	0.93 (0.75–1.15)	0.79 (0.56–1.10)
	3	1.01 (0.97–1.05)	1.13 (0.96–1.34)	1.11 (0.65–1.91)	1.07 (0.94–1.21)	1.08 (0.87–1.33)	0.70 (0.49–0.99)
	4	0.97 (0.94–1.01)	1.00 (0.85–1.17)	1.48 (0.92–2.38)	0.94 (0.83–1.06)	0.95 (0.77–1.16)	0.60 (0.43–0.84)
	5	1.04 (1.01–1.07)	1.03 (0.89–1.20)	1.29 (0.81–2.04)	0.92 (0.82–1.04)	0.93 (0.77–1.13)	0.49 (0.36–0.67)
	6	1.04 (1.01–1.08)	1.01 (0.87–1.18)	1.85 (1.18–2.89)	0.99 (0.88–1.11)	1.11 (0.92–1.33)	0.55 (0.40–0.74)
	7	1.04 (1.01–1.07)	0.90 (0.78–1.03)	1.63 (1.06–2.52)	0.89 (0.79–0.99)	1.07 (0.90–1.28)	0.43 (0.33–0.58)
	8	1.05 (1.02–1.08)	1.02 (0.88–1.18)	2.75 (1.79–4.23)	1.03 (0.93–1.15)	1.04 (0.87–1.24)	0.53 (0.39–0.71)
	9	1.04 (1.01–1.07)	1.00 (0.87–1.15)	1.91 (1.25–2.93)	0.97 (0.87–1.08)	0.92 (0.77–1.09)	0.27 (0.20–0.36)
	10 (richest)	1.06 (1.03–1.09)	1.16 (1.01–1.32)	2.01 (1.32–3.06)	1.06 (0.96–1.18)	1.12 (0.95–1.33)	0.27 (0.20–0.35)
Region*							
	Northeast	1.00	1.00	1.00	1.00	1.00	1.00
	North	0.97 (0.95–0.98)	1.15 (1.07–1.23)	0.89 (0.74–1.08)	0.78 (0.73–0.82)	0.46 (0.41–0.51)	0.60 (0.51–0.72)
	Midwest	1.04 (1.02–1.05)	1.17 (1.07–1.26)	2.12 (1.79–2.50)	0.96 (0.91–1.03)	0.94 (0.86–1.03)	0.47 (0.37–0.60)
	South	1.04 (1.04–1.05)	1.33 (1.24–1.42)	1.63 (1.39–1.91)	1.03 (0.98–1.09)	0.70 (0.64–0.76)	0.40 (0.32–0.50)
	Southeast	1.03 (1.03–1.04)	1.27 (1.21–1.34)	1.62 (1.43–1.85)	1.07 (1.03–1.12)	0.95 (0.90–1.01)	0.40 (0.35–0.46)

* Values adjusted for gender and age; ICU: intensive care unit.

Case fatality rate also varied according to the macro-region of residence. Compared to the Northeast region, the case fatality rate in children was significantly lower in all other regions. Among adolescents, the case fatality rate was lower in the Midwest Region. As to other outcomes, the North Region had the lowest numbers for biological samples collected and patients admitted to ICU beds (Table 2 and Supplementary Table 2).

**Figure 1:**
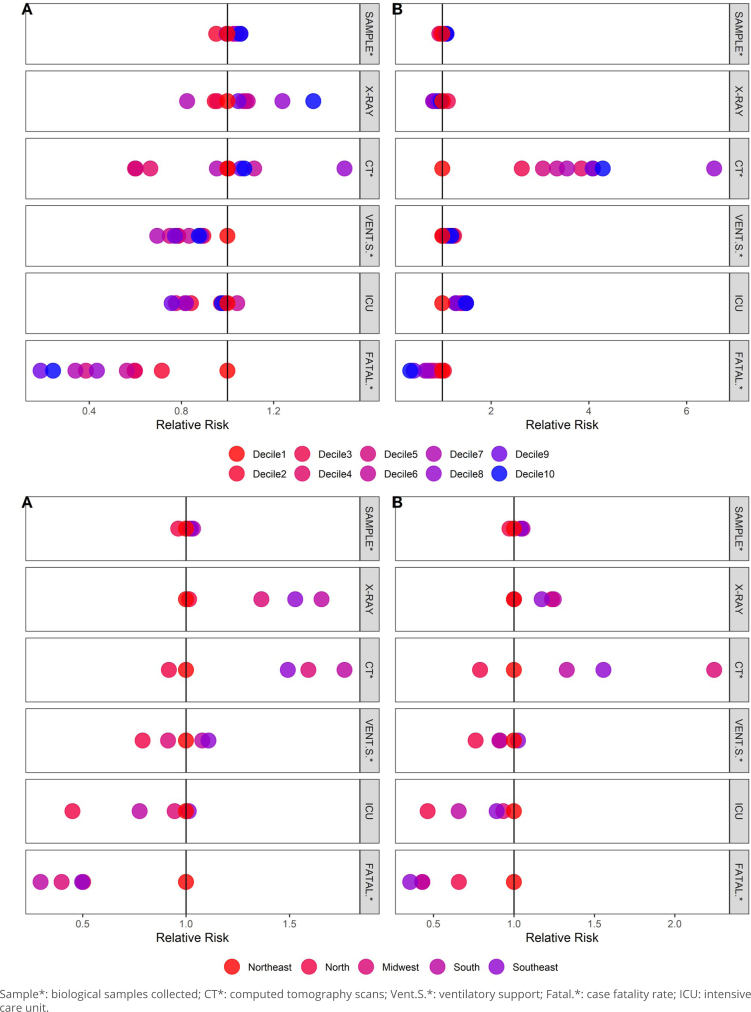
Relative risk adjusted for gender and age of biological samples collected, X-rays, computed tomography scans, ventilatory support, intensive care unit hospitalization, and case fatality rate in children in the (A) first and (B) second waves of the COVID-19 pandemic, according to gross domestic product *per capita* deciles of municipalities and macro-regions. Brazil, March 2020 to December 2021.

The directions of the associations in the two years analyzed were the same when considering the two COVID-19 waves characterized in the study separately, but with some variation in the magnitude of the differences. While the case fatality rate for the first wave was four times higher among children living in municipalities with lower GDP *per capita*, the value for the second wave was almost three times higher (Figure 1). No statistically significant differences were found in the case fatality rate in the first wave among adolescents, even though richer municipalities presented fewer specific measures. In the second wave, though, the case fatality rate was twice as high in municipalities with lower GDP *per capita* (Supplementary Figure 1).

As for the remaining outcomes in children, residents in the richer municipality decile presented a higher probability of having their biological samples collected to diagnose COVID-19 in both the first (RR=1.06; 95%CI 1.01–1.11) and second (RR=1.09; 95%CI 1.03–1.14) waves (Figure 1). X-ray examinations were 37% more frequent in these municipalities in the first wave (RR=1.37; 95%CI 1.09–1.72). Regarding the number of CT scans, we found no statistically significant differences in the first wave; however, the event was four times higher (RR=4.29; 95%CI 1.8–10.19) in the second wave in residents of richer municipalities (Figure 1). In adolescents, the proportion of patients who underwent a CT scan was twice as high in richer municipalities compared to poorer ones in the first wave and three times as high in the second wave (Supplementary Figure 1).

The macro-regions showed differences in both COVID-19 waves. Compared to the Northeast Region, the lowest case fatality rate in children was found in the South Region (RR=0.30; 95%CI 0.18–0.50) in the first wave and in the Southeast Region (RR=0.36; 95%CI 0.29–0.44) in the second wave. The South, Southeast, and Midwest regions had more biological samples collected and CT scans in both waves among children (Figure 1). Among adolescents, the lowest case fatality rate was found in the Midwest Region both in the first (RR=0.57; 95%CI 0.33–0.99) and second (RR=0.40; 95%CI 0.25–0.64) waves compared to the Northeast Region (Supplementary Figure 1).

## DISCUSSION

This study identified inequalities in the hospital case fatality rate of COVID-19 and hospital care among pediatric and adolescent patients with the disease according to the GDP *per capita* of municipalities and regions of residence. Municipalities with lower GDP *per capita* presented higher hospital case fatality rates, fewer biological samples collected to diagnose the infectious agent, and fewer X-ray examinations and CT scans. We found regional disparities associated with case fatality rates in this specific population, with worse indicators in the North and Northeast regions. In general, the findings remained consistent over the two COVID-19 waves analyzed.

The hospital case fatality rate found in the sample was higher than that reported in previous pediatric studies^14,26,27^. A study from the United Kingdom revealed that six (1%) out of the 627 pediatric patients hospitalized for COVID-19 followed died in the hospital^27^. Studies carried out in United States hospitals showed a case fatality rate of around 2%^14,26^.

The differences in unfavorable results between this study and those from other countries may be related to the policy adopted to combat the pandemic and the fact that Brazil has important socioeconomic inequalities that may affect the quality and availability of health services in certain regions and to certain groups, increasing the likelihood of worse clinical outcomes^28^. The greater case fatality rate identified in municipalities with a lower GDP *per capita* corroborates previous findings, with mortality almost four times higher in children living in more disadvantaged municipalities in Mexico^3^ and Brazil^23^, as well as higher case fatality rates in the North and Northeast regions^1,4,23,29^. Investigations conducted in the United States indicated that patients from lower-income neighborhoods or municipalities were more likely to die^30,31^; however, this relationship is still scarcely studied in children.

The literature has shown that social and health inequalities result in unfavorable COVID-19 prognosis, be it in high- or low-income countries, or in adult or pediatric populations^32,33^. These data reinforce the hypothesis that pandemics affect populations unequally, as health access is usually lower in less-favored regions, with variations in diagnosis and access to ICU beds, even in universal health systems, such as the one in Brazil^34^. Previous publications revealed that the health services provided in the North and Northeast regions are more concentrated in capitals and a few regional centers of metropolitan areas. On the other hand, the health system is better distributed within states in the South and Southeast regions^23,35^. Poorer populations and municipalities, which respectively already have more difficulty accessing and providing health services under normal circumstances, are the most vulnerable in times of crisis and should be prioritized in action planning, something that did not occur in Brazil during the COVID-19 pandemic^35^.

Health inequalities can influence the results of the COVID-19 pandemic. Despite the importance of testing for SARS-CoV-2 infection control, the strategies adopted differed between and within countries^36^. Some countries adopted broader strategies, testing suspected and symptomatic cases. Others tested only severely symptomatic people, those who had contact with a confirmed case, and individuals belonging to risk groups^36^. Brazil did not implement a broad and structured testing policy. In the present study, poorer municipalities had fewer biological samples collected for diagnosis, which may indicate a lower testing capacity in these locations, contributing to the lack of control of the pandemic, with the consequent collapse of health services and increased pediatric mortality.

Studies in Europe and the United States investigating the general population showed that test resources were disproportionately distributed among the population according to the place of residence and were more accessible in richer regions^37,38^. Another publication from the United States^39^ identified an unequal test distribution, evidencing pre-existing structural disparities, since test sites were implemented in already built infrastructures, exacerbating discrepancies in geographical access. In Brazil, besides an insufficient testing capacity for the demand, the federal response was uncoordinated^40,41^, increasing regional disparities in health care. In a context of great inequality, the lack of federal planning resulted in states and municipalities competing to acquire resources^35^. Thus, the response capacity of each state and the Federal District was clearly different, as they had unequal conditions for action^35^.

Another important finding was the lower number of imaging tests performed in poorer municipalities. A systematic review that included mainly Italian, American, and Chinese children showed that 73.9% of them and 64% of newborns were submitted to CT scans^42^. An American study indicated that X-rays were performed in 72% of children admitted to a hospital in New York City, NY, United States^26^. Imaging tests may be indicated for diagnosis in the absence of RT-PCR tests or used to evaluate disease stages and severity^43,44^. Given the lack of indication for their universal use in COVID-19 cases, the differences observed may be due to different clinical protocols between municipalities or, more likely, be associated with disparities in the physical structure of the hospital and/or the availability of specialized professionals in these locations.

We underline that CT scans require highly specialized equipment that may not be available in poorer Brazilian municipalities and regions. Therefore, access to this test may be limited to larger cities, evidencing the health system's inability to provide the highest level of care to severe patients^34^, a situation that may have contributed to the results found in this study. Previous publications showed that a large part of the 450 health districts defined by the Brazilian public health system does not have the necessary resources to respond to a pandemic^45,46^. Still, these results should be analyzed with caution since CT scans expose patients to radiation and health professionals to a higher risk of cross-contamination within the hospital^47,48^. The American College of Radiology advised against using this examination as a primary diagnostic method^47^. Thus, the lack of specific guidelines for pediatric cases infected with the novel coronavirus can contribute to its use on a smaller or larger scale.

The results indicate that COVID-19 affected the pediatric population in Brazil based on patterns of socioeconomic vulnerability, exacerbating pre-existing inequalities and producing worse results in poorer regions and municipalities. These results could have been mitigated by a government response tailored to local needs. However, without a coordinated national strategy, the responses were heterogeneous^49^. Because the Brazilian health system is decentralized, local governments could implement their own public health interventions and adopt measures to increase hospital capacity. Yet, without national coordination and equitable funding, richer municipalities had more action options and better structural conditions prior to the pandemic.

In this scenario, we emphasize that COVID-19 resources and care were directed toward age groups affected by the disease in greater numbers, without paying more careful attention to the needs of children and adolescents. We should remember that, in addition to COVID-19 mortality, the repercussions of the pandemic on child health can lead children to a higher risk of morbidity and mortality due to preventable diseases, given the discontinuity of vaccination and other health care routines. Another latent concern is the suspension of school activities, increasing the quality disparity between public and private education and harming those who need it the most^50^.

The strength of the present study was the analysis of an expressive number of cases, which was possible thanks to the use of SIVEP-Gripe, a large nationwide database whose filling is compulsory for public and private health services, allowing us to collect COVID-19 epidemiological data from hospitalized children and adolescents. Still, the study has limitations. The analysis may have been affected by inadequate data filling and failure to enter cases into the system. Underreporting is also a likely problem, especially in more vulnerable socioeconomic contexts. Thus, even restricted to estimating the in-hospital case fatality rate, we highlight that the case fatality rate of COVID-19 in patients who required more intensive care and could not access the health facility may be greater outside the hospital.

Since this study analyzed hospitalized patients, the sample tends to represent only individuals at the most severe end of the disease spectrum, and this aspect should be considered when interpreting the data. This study did not investigate the presence of pre-existing diseases or other covariates that differentiate municipalities among themselves. Moreover, the study did not aim to examine factors that mediate the association of macrosocial characteristics with the case fatality rate of COVID-19 and care outcomes related to the disease. Further research exploring aspects related to housing and working conditions, the health care system, and the previous burden of diseases will be important to broaden the understanding of the associations described herein.

In this study, socioeconomic and regional vulnerabilities were associated with the case fatality rate and care of children and adolescents hospitalized for COVID-19 in Brazil. We found a higher risk of death among residents of municipalities with lower GDP *per capita* and of the North and Northeast regions. These results are important for the formulation of social policies that consider the characteristics of the population when combating COVID-19 so as to promote equitable health.
