# Supplementary material for: Socioeconomic inequality in hospital case fatality rate and care among children and adolescents hospitalized for COVID-19 in Brazil
Source: Rev Bras Epidemiol. 2023 Feb 20;26:e230015. doi: 10.1590/1980-549720230015 (PMC9949490; doi:10.1590/1980-549720230015)
Supplement: Supplementary file 1 [file 1980-5497-rbepid-26-e230015-Suppl01.pdf]

Tabela suplementar 1: Descrição da amostra, proporção de coleta de amostra biológica, realização de raio-x, tomografia, uso de suporte ventilatório, internações em UTI e a letalidade de adolescentes internados por Covid-19, segundo os decis do Produto Interno Bruto dos municípios e a macrorregião. Brasil, março de 2020 - dezembro de 2021.

	Amostra n	Coleta de amostra biológica %	Realização de raio-x %	Realização de tomografia %	Uso de suporte ventilatório %	Internação em UTI %	Letalidade %
Decil de renda							
1 (+ pobres)	148	94,6	20,9	16,2	51,4	29,1	16,2
2	207	91,3	29,5	14,5	54,1	26,6	13,5
3	179	95,0	27,4	23,5	55,3	27,4	16,2
4	230	93,9	25,7	20,9	51,7	30,0	14,8
5	367	95,9	22,6	22,3	52,9	26,7	11,7
6	401	95,5	22,9	30,7	55,1	34,7	14,2
7	787	94,7	19,4	27,3	54,8	29,1	13,1
8	589	96,1	23,9	35,5	58,6	29,7	9,7
9	1150	96,3	23,1	35,0	59,3	27,8	11,2
10 (+ ricos)	2269	97,9	27,2	39,0	56,1	32,2	8,8

Região							
Nordeste	1165	95,5	18,4	19,1	51,5	33,6	15,2
Norte	820	92,1	26,1	16,8	49,4	17,9	12,8
Centro-Oeste	676	95,1	16,1	48,8	55,8	31,5	8,0
Sul	1179	98,6	23,2	40,1	60,3	26,1	10,1
Sudeste	2487	97,2	29,8	36,1	58,7	34,1	10,0
Total da amostra	6327	96,3	24,5	32,6	56,1	30,1	11,1

Tabela suplementar 2: Risco relativo da coleta de amostra biológica, realização de raio-x, tomografia, uso de suporte ventilatório, internações em UTI e letalidade entre adolescentes internados por Covid-19, segundo os decis do Produto Interno Bruto per capita dos municípios e a macrorregião. Brasil, março de 2020 - dezembro de 2021.

	Coleta de amostra biológica (IC 95%)	Realização de raio-x (IC 95%)	Realização de tomografia (IC 95%)	Uso de suporte ventilatório (IC 95%)	Internação em UTI (IC 95%)	Letalidade (IC 95%)
Decil de renda*						
1(+pobres)	1,00	1,00	1,00	1,00	1,00	1,00
2	0,96 (0,91-1,02)	1,36 (0,93-1,98)	0,89 (0,54-1,45)	1,03 (0,84-1,26)	0,89 (0,63-1,25)	0,80 (0,48-1,33)
3	1,00 (0,95-1,06)	1,33 (0,90-1,96)	1,39 (0,89-2,18)	1,04 (0,85-1,28)	0,93 (0,66-1,32)	0,95 (0,58-1,55)
4	0,99 (0,94-1,04)	1,17 (0,80-1,72)	1,29 (0,83-2,01)	0,99 (0,81-1,21)	1,00 (0,73-1,38)	0,89 (0,55-1,43)
5	1,01 (0,97-1,06)	1,07 (0,75-1,54)	1,36 (0,90-2,06)	1,02 (0,85-1,22)	0,91 (0,67-1,23)	0,71 (0,45-1,12)
6	1,01 (0,97-1,06)	1,07 (0,75-1,53)	1,86 (1,25-2,75)	1,04 (0,87-1,25)	1,16 (0,88-1,55)	0,84 (0,54-1,30)
7	1,00 (0,96-1,04)	0,91 (0,65-1,28)	1,67 (1,14-2,44)	1,05 (0,88-1,24)	0,98 (0,75-1,29)	0,78 (0,52-1,18)
8	1,02 (0,97-1,06)	1,12 (0,80-1,58)	2,13 (1,46-3,12)	1,11 (0,94-1,32)	1,00 (0,76-1,32)	0,57 (0,37-0,89)
9	1,02 (0,98-1,06)	1,09 (0,78-1,51)	2,11 (1,45-3,06)	1,12 (0,95-1,32)	0,94 (0,72-1,23)	0,66 (0,44-0,99)
10(+ricos)	1,04 (1,00-1,08)	1,26 (0,92-1,73)	2,37 (1,64-3,43)	1,06 (0,91-1,25)	1,08 (0,83-1,39)	0,52 (0,35-0,76)

Região*						
Nordeste	1,00	1,00	1,00	1,00	1,00	1,00
Norte	0,96 (0,94-0,99)	1,42 (1,20-1,68)	0,88 (0,73-1,07)	0,96 (0,88-1,05)	0,53 (0,45-0,63)	0,84 (0,67-1,05)
Centro-Oeste	1,00 (0,98-1,02)	0,89 (0,72-1,10)	2,53 (2,19-2,91)	1,08 (0,99-1,18)	0,94 (0,82-1,08)	0,52 (0,39-0,70)
Sul	1,03 (1,02-1,05)	1,27 (1,08-1,48)	2,07 (1,81-2,38)	1,16 (1,08-1,25)	0,77 (0,68-0,88)	0,65 (0,53-0,81)
Sudeste	1,02 (1,00-1,03)	1,62 (1,41-1,85)	1,87 (1,64-2,13)	1,13 (1,06-1,21)	1,01 (0,91-1,11)	0,65 (0,54-0,78)

* Valores ajustados por sexo e idade

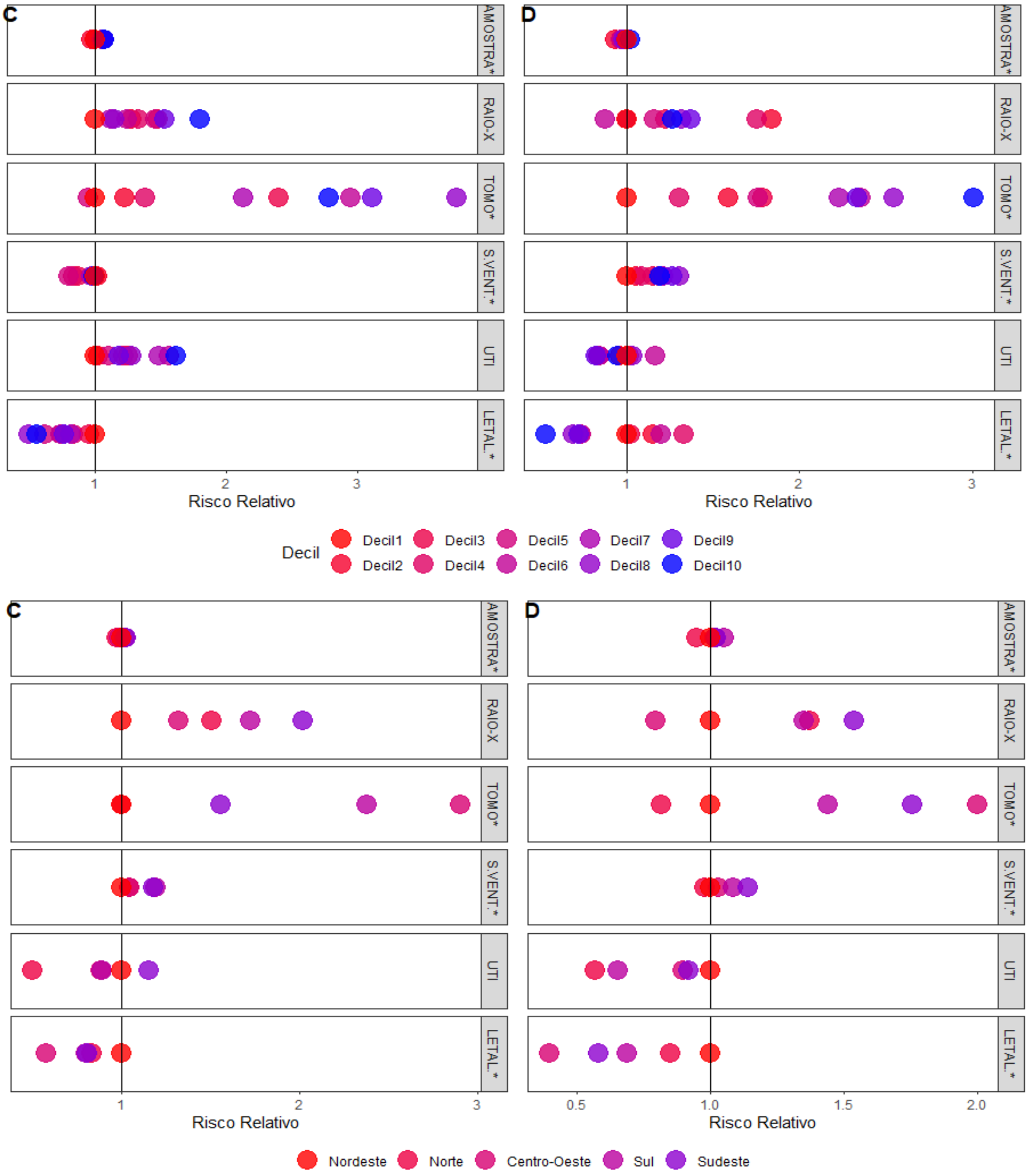

Amostra: Coleta de amostra biológica; Tomo: Exame de tomografia. *S.Vent.: Uso de suporte ventilatório. Letal*: Letalidade

Figura suplementar 1: Risco relativo ajustado por sexo e idade da coleta de amostra biológica, realização de raio-x, tomografia, uso de suporte ventilatório, internação em UTI e letalidade em adolescentes durante a primeira (C) e segunda onda (D), de acordo com os decis do Produto Interno Bruto dos municípios e com a macrorregião. Brasil, marco de 2020 - dezembro de 2021.

Tabela suplementar 3: Ponto de corte do Produto Interno Bruto per capita dos municípios brasileiros de acordo com o decil de renda.

Decil de renda	Ponto de Corte PIB (R$)
1(+pobres)	7.695.123,00
2	9.050.378,00
3	10.927.778,00
4	13.849.310,00
5	17.433.840,00
6	21.287.970,00
7	25.844.532,00
8	32.335.742,00
9	44.325.452,00
10(+ricos)	58.317.185,00
